# 4-Nitro-*N*-[(*E*)-thio­phen-2-yl­methyl­idene]aniline

**DOI:** 10.1107/S1600536812028346

**Published:** 2012-06-30

**Authors:** Abdullah M. Asiri, Hassan M. Faidallah, Seik Weng Ng, Edward R. T. Tiekink

**Affiliations:** a Chemistry Department, Faculty of Science, King Abdulaziz University, PO Box 80203, Jeddah, Saudi Arabia; b Department of Chemistry, University of Malaya, 50603 Kuala Lumpur, Malaysia

## Abstract

In the title compound, C_11_H_8_N_2_O_2_S, there is a twist in the mol­ecule, with the dihedral angle between the five- and six-membered rings being 31.77 (9)°. The nitro group is slightly twisted out of the plane of the benzene ring to which it is attached [O—N—C—C torsion angle = 9.0 (3)°]. The S and N atoms are *syn*. In the crystal, supra­molecular layers parallel to (-204) are formed by C—H⋯O and C—H⋯N inter­actions. These layers are connected into a three-dimensional architecture by π–π inter­actions occurring between centrosymmetrically related benzene rings [centroid–centroid distance = 3.6020 (11) Å].

## Related literature

For background to 2-substituted thio­phenes, see: Kleemann *et al.* (2006 ▶). For a related structure, see: Asiri *et al.* (2012 ▶).
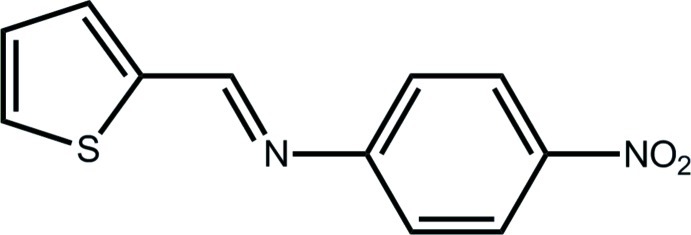


## Experimental

### 

#### Crystal data

C_11_H_8_N_2_O_2_S
*M* *_r_* = 232.25
Monoclinic, 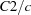

*a* = 9.2754 (5) Å
*b* = 11.9983 (9) Å
*c* = 18.4996 (13) Å
β = 92.772 (6)°
*V* = 2056.4 (2) Å^3^
*Z* = 8
Mo *K*α radiation
μ = 0.30 mm^−1^
*T* = 100 K
0.25 × 0.15 × 0.05 mm

#### Data collection

Agilent SuperNova Dual diffractometer with an Atlas detector
Absorption correction: multi-scan (*CrysAlis PRO*; Agilent, 2012 ▶) *T*_min_ = 0.692, *T*_max_ = 1.000
8697 measured reflections
2377 independent reflections
1869 reflections with *I* > 2σ(*I*)
*R*_int_ = 0.050

#### Refinement

*R*[*F* ^2^ > 2σ(*F* ^2^)] = 0.040
*wR*(*F* ^2^) = 0.111
*S* = 1.04
2377 reflections
145 parameters
H-atom parameters constrained
Δρ_max_ = 0.29 e Å^−3^
Δρ_min_ = −0.31 e Å^−3^

### 

Data collection: *CrysAlis PRO* (Agilent, 2012 ▶); cell refinement: *CrysAlis PRO*; data reduction: *CrysAlis PRO*; program(s) used to solve structure: *SHELXS97* (Sheldrick, 2008 ▶); program(s) used to refine structure: *SHELXL97* (Sheldrick, 2008 ▶); molecular graphics: *ORTEP-3* (Farrugia, 1997 ▶) and *DIAMOND* (Brandenburg, 2006 ▶); software used to prepare material for publication: *publCIF* (Westrip, 2010 ▶).

## Supplementary Material

Crystal structure: contains datablock(s) global, I. DOI: 10.1107/S1600536812028346/bt5953sup1.cif

Structure factors: contains datablock(s) I. DOI: 10.1107/S1600536812028346/bt5953Isup2.hkl

Supplementary material file. DOI: 10.1107/S1600536812028346/bt5953Isup3.cml

Additional supplementary materials:  crystallographic information; 3D view; checkCIF report

## Figures and Tables

**Table 1:** Hydrogen-bond geometry (Å, °)

*D*—H⋯*A*	*D*—H	H⋯*A*	*D*⋯*A*	*D*—H⋯*A*
C1—H1⋯O2^i^	0.95	2.50	3.412 (2)	160
C2—H2⋯N1^ii^	0.95	2.62	3.556 (2)	169

Symmetry codes: (i) 
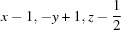
; (ii) 
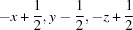
.

**Fig. 1.**
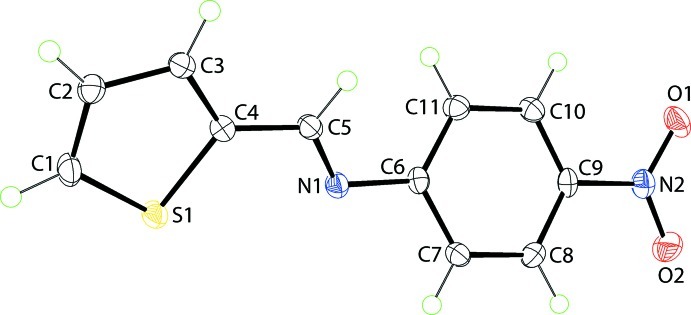
The molecular structure of (I) showing the atom-labelling scheme and displacement ellipsoids at the 50% probability level.

**Fig. 2.**
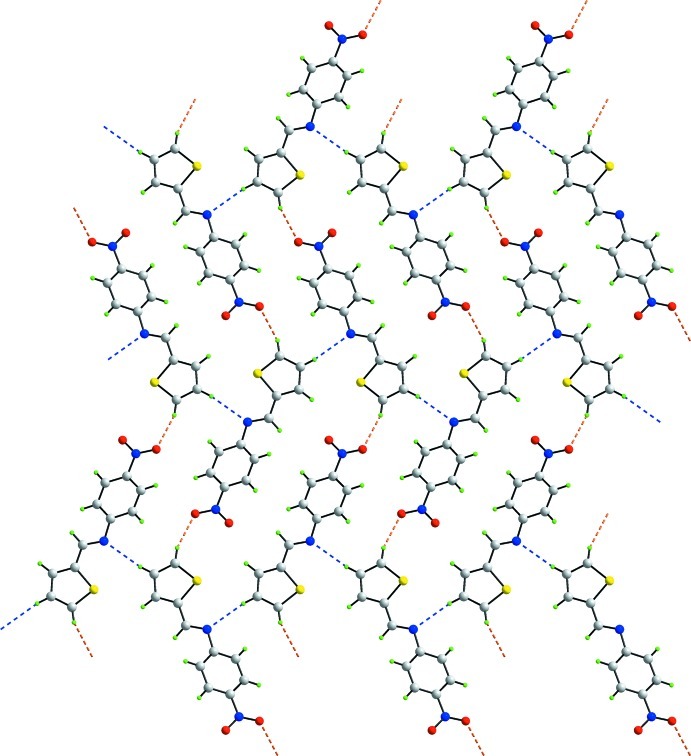
A view of the supramolecular layer in the (-2 0 4) plane in (I) mediated by C—H···O and C—H···N interactions shown as orange and blue dashed lines, respectively.

**Fig. 3.**
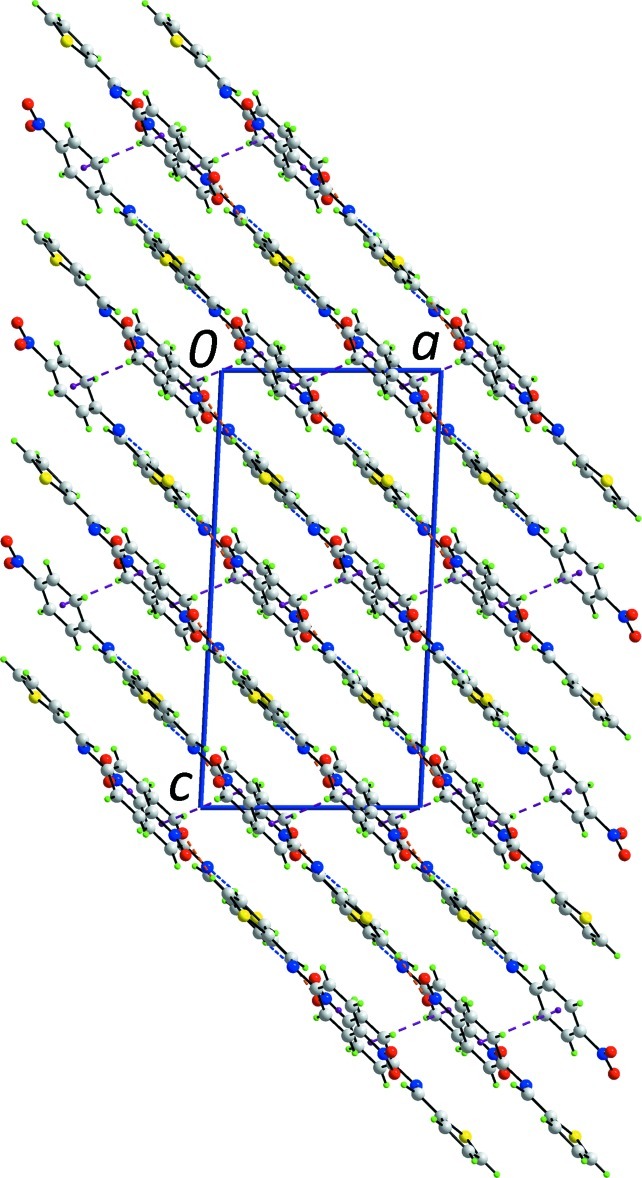
A view in projection down the b axis of the unit-cell contents of (I), showing the stacking of supramolecular layers. The C—H···O, C—H···N and C—H—π interactions are shown as orange, blue and purple dashed lines, respectively.

## supplementary crystallographic information

### Comment

Among the various useful properties exhibited by thiophenes are their biological activities (Kleemann *et al.*, 2006). In continuation of structural studies of thienyl derivatives (Asiri *et al.*, 2012), herein the crystal structure determination of the title compound, (4-nitrophenyl)thiophene-2-ylmethylene-amine (I), is described.

In (I), Fig. 1, the conformation about the N1═C5 bond [1.283 (2) Å] is *E*. A twist in the molecule is evident as seen in the value of the dihedral angle of 31.77 (9)° between the five- and six-membered rings. The major deviation from a planar torsion angle is -37.0 (3)° for C5—N1—C6—C11 indicating that the most significant twist occurs around the N1—C6 bond. The nitro group is slightly inclined with respect to the plane of the benzene ring to which it is attached as seen in the O2—N2—C9—C8 torsion angle of 9.0 (3)°. The S and N atoms are *syn*.

In the crystal packing, supramolecular layers parallel to (-2 0 4) are formed by C—H···O and C—H···N interactions, Table 1, which lead to 22-membered {···HC_2_H···ONC_4_N}_2_ synthons (Fig 2). Layers aggregate to form a three-dimensional architecture by π—π interactions occurring between centrosymmetrically related benzene rings [inter-centroid distance = 3.6020 (11) Å for symmetry operation 1 - *x*, 1 - *y*, 1 - *z*], Fig. 3.

### Experimental

A mixture of thiophen-2-carboxaldehyde (1.1 g, 0.01 *M*) and *p*-nitroaniline (1.4 g, 0.0 1*M*) in ethanol (10 ml) was heated on a water bath for 30 min, the solid which separated out was filtered, dried and recrystallized from ethanol as yellow prisms; *M*. pt: 375–376 K. Yield: 96%.

### Refinement

Carbon-bound H-atoms were placed in calculated positions [C—H = 0.95 Å, *U*_iso_(H) = 1.2*U*_eq_(C)] and were included in the refinement in the riding model approximation.

### Crystal data

**Table:**

C_11_H_8_N_2_O_2_S	*F*(000) = 960
*M**_r_* = 232.25	*D*_x_ = 1.500 Mg m^−^^3^
Monoclinic, *C*2/*c*	Mo *K*α radiation, λ = 0.71073 Å
Hall symbol: -C 2yc	Cell parameters from 2057 reflections
*a* = 9.2754 (5) Å	θ = 2.8–27.5°
*b* = 11.9983 (9) Å	µ = 0.30 mm^−^^1^
*c* = 18.4996 (13) Å	*T* = 100 K
β = 92.772 (6)°	Prism, yellow
*V* = 2056.4 (2) Å^3^	0.25 × 0.15 × 0.05 mm
*Z* = 8	

### Data collection

**Table:**

Agilent SuperNova Dual diffractometer with an Atlas detector	2377 independent reflections
Radiation source: SuperNova (Mo) X-ray Source	1869 reflections with *I* > 2σ(*I*)
Mirror monochromator	*R*_int_ = 0.050
Detector resolution: 10.4041 pixels mm^-1^	θ_max_ = 27.6°, θ_min_ = 2.8°
ω scan	*h* = −11→12
Absorption correction: multi-scan (*CrysAlis PRO*; Agilent, 2012)	*k* = −13→15
*T*_min_ = 0.692, *T*_max_ = 1.000	*l* = −22→24
8697 measured reflections	

### Refinement

**Table:**

Refinement on *F*^2^	Primary atom site location: structure-invariant direct methods
Least-squares matrix: full	Secondary atom site location: difference Fourier map
*R*[*F*^2^ > 2σ(*F*^2^)] = 0.040	Hydrogen site location: inferred from neighbouring sites
*wR*(*F*^2^) = 0.111	H-atom parameters constrained
*S* = 1.04	*w* = 1/[σ^2^(*F*_o_^2^) + (0.049*P*)^2^ + 1.4742*P*] where *P* = (*F*_o_^2^ + 2*F*_c_^2^)/3
2377 reflections	(Δ/σ)_max_ < 0.001
145 parameters	Δρ_max_ = 0.29 e Å^−^^3^
0 restraints	Δρ_min_ = −0.31 e Å^−^^3^

### Special details

**Table:**

Geometry. All e.s.d.'s (except the e.s.d. in the dihedral angle between two l.s. planes) are estimated using the full covariance matrix. The cell e.s.d.'s are taken into account individually in the estimation of e.s.d.'s in distances, angles and torsion angles; correlations between e.s.d.'s in cell parameters are only used when they are defined by crystal symmetry. An approximate (isotropic) treatment of cell e.s.d.'s is used for estimating e.s.d.'s involving l.s. planes.
Refinement. Refinement of *F*^2^ against ALL reflections. The weighted *R*-factor *wR* and goodness of fit *S* are based on *F*^2^, conventional *R*-factors *R* are based on *F*, with *F* set to zero for negative *F*^2^. The threshold expression of *F*^2^ > σ(*F*^2^) is used only for calculating *R*-factors(gt) *etc*. and is not relevant to the choice of reflections for refinement. *R*-factors based on *F*^2^ are statistically about twice as large as those based on *F*, and *R*- factors based on ALL data will be even larger.

### Fractional atomic coordinates and isotropic or equivalent isotropic displacement parameters (Å^2^)

**Table:**

	*x*	*y*	*z*	*U*_iso_*/*U*_eq_	
S1	0.22121 (5)	0.35279 (4)	0.24739 (3)	0.01985 (16)	
O1	0.94206 (15)	0.49221 (12)	0.60866 (8)	0.0287 (4)	
O2	0.92261 (17)	0.65565 (13)	0.55892 (8)	0.0330 (4)	
N1	0.45609 (16)	0.40163 (13)	0.36388 (8)	0.0172 (3)	
N2	0.88841 (17)	0.55649 (14)	0.56311 (9)	0.0213 (4)	
C1	0.1448 (2)	0.24635 (16)	0.19692 (10)	0.0216 (4)	
H1	0.0693	0.2567	0.1610	0.026*	
C2	0.2041 (2)	0.14558 (16)	0.21402 (10)	0.0191 (4)	
H2	0.1742	0.0776	0.1916	0.023*	
C3	0.3151 (2)	0.15307 (15)	0.26874 (10)	0.0178 (4)	
H3	0.3687	0.0909	0.2871	0.021*	
C4	0.33686 (19)	0.26104 (15)	0.29257 (10)	0.0165 (4)	
C5	0.44640 (19)	0.29889 (16)	0.34505 (10)	0.0168 (4)	
H5	0.5128	0.2465	0.3661	0.020*	
C6	0.56731 (19)	0.43424 (15)	0.41452 (10)	0.0156 (4)	
C7	0.6258 (2)	0.54040 (16)	0.40547 (10)	0.0186 (4)	
H7	0.5925	0.5852	0.3658	0.022*	
C8	0.7316 (2)	0.58087 (16)	0.45373 (10)	0.0195 (4)	
H8	0.7716	0.6530	0.4477	0.023*	
C9	0.77767 (19)	0.51350 (16)	0.51104 (9)	0.0172 (4)	
C10	0.7220 (2)	0.40827 (16)	0.52155 (10)	0.0186 (4)	
H10	0.7565	0.3637	0.5612	0.022*	
C11	0.6151 (2)	0.36890 (16)	0.47345 (10)	0.0190 (4)	
H11	0.5742	0.2974	0.4805	0.023*	

### Atomic displacement parameters (Å^2^)

**Table:**

	*U*^11^	*U*^22^	*U*^33^	*U*^12^	*U*^13^	*U*^23^
S1	0.0209 (3)	0.0158 (3)	0.0223 (3)	0.00164 (19)	−0.00515 (19)	−0.00144 (18)
O1	0.0258 (8)	0.0328 (9)	0.0264 (8)	0.0027 (7)	−0.0107 (6)	0.0029 (6)
O2	0.0361 (9)	0.0281 (9)	0.0334 (9)	−0.0135 (7)	−0.0118 (7)	0.0019 (7)
N1	0.0164 (8)	0.0180 (8)	0.0169 (8)	−0.0001 (6)	−0.0022 (6)	−0.0005 (6)
N2	0.0182 (8)	0.0258 (10)	0.0196 (9)	−0.0014 (7)	−0.0017 (7)	−0.0019 (7)
C1	0.0191 (10)	0.0246 (11)	0.0203 (10)	−0.0014 (8)	−0.0060 (8)	−0.0028 (8)
C2	0.0213 (10)	0.0182 (10)	0.0175 (10)	−0.0024 (8)	−0.0015 (8)	−0.0021 (7)
C3	0.0190 (9)	0.0158 (10)	0.0183 (9)	0.0004 (7)	−0.0019 (7)	0.0011 (7)
C4	0.0160 (9)	0.0173 (9)	0.0160 (9)	0.0010 (7)	−0.0002 (7)	0.0003 (7)
C5	0.0161 (9)	0.0183 (10)	0.0161 (9)	0.0014 (8)	−0.0002 (7)	0.0023 (7)
C6	0.0133 (8)	0.0174 (9)	0.0160 (9)	0.0012 (7)	−0.0003 (7)	−0.0033 (7)
C7	0.0176 (9)	0.0203 (10)	0.0176 (9)	0.0011 (8)	−0.0008 (7)	0.0040 (7)
C8	0.0189 (9)	0.0181 (10)	0.0214 (10)	−0.0040 (8)	−0.0003 (8)	0.0006 (7)
C9	0.0124 (9)	0.0243 (10)	0.0148 (9)	−0.0006 (7)	−0.0009 (7)	−0.0035 (7)
C10	0.0202 (9)	0.0190 (10)	0.0165 (9)	0.0016 (8)	−0.0014 (7)	0.0008 (7)
C11	0.0220 (10)	0.0165 (9)	0.0184 (10)	−0.0003 (8)	−0.0001 (8)	−0.0005 (7)

### Geometric parameters (Å, º)

**Table:**

S1—C1	1.7150 (19)	C4—C5	1.444 (2)
S1—C4	1.7246 (18)	C5—H5	0.9500
O1—N2	1.230 (2)	C6—C11	1.398 (3)
O2—N2	1.235 (2)	C6—C7	1.397 (3)
N1—C5	1.283 (2)	C7—C8	1.382 (3)
N1—C6	1.415 (2)	C7—H7	0.9500
N2—C9	1.467 (2)	C8—C9	1.384 (3)
C1—C2	1.359 (3)	C8—H8	0.9500
C1—H1	0.9500	C9—C10	1.382 (3)
C2—C3	1.411 (3)	C10—C11	1.383 (3)
C2—H2	0.9500	C10—H10	0.9500
C3—C4	1.380 (3)	C11—H11	0.9500
C3—H3	0.9500		
			
C1—S1—C4	91.13 (9)	C4—C5—H5	119.3
C5—N1—C6	119.00 (16)	C11—C6—C7	119.63 (17)
O1—N2—O2	123.41 (16)	C11—C6—N1	123.71 (17)
O1—N2—C9	118.47 (16)	C7—C6—N1	116.58 (16)
O2—N2—C9	118.12 (16)	C8—C7—C6	120.68 (17)
C2—C1—S1	112.55 (15)	C8—C7—H7	119.7
C2—C1—H1	123.7	C6—C7—H7	119.7
S1—C1—H1	123.7	C7—C8—C9	118.21 (17)
C1—C2—C3	112.54 (17)	C7—C8—H8	120.9
C1—C2—H2	123.7	C9—C8—H8	120.9
C3—C2—H2	123.7	C10—C9—C8	122.53 (17)
C4—C3—C2	112.28 (17)	C10—C9—N2	118.91 (16)
C4—C3—H3	123.9	C8—C9—N2	118.56 (17)
C2—C3—H3	123.9	C9—C10—C11	118.88 (18)
C3—C4—C5	126.67 (18)	C9—C10—H10	120.6
C3—C4—S1	111.50 (14)	C11—C10—H10	120.6
C5—C4—S1	121.73 (14)	C10—C11—C6	120.05 (18)
N1—C5—C4	121.49 (17)	C10—C11—H11	120.0
N1—C5—H5	119.3	C6—C11—H11	120.0
			
C4—S1—C1—C2	0.33 (16)	N1—C6—C7—C8	177.85 (16)
S1—C1—C2—C3	−0.5 (2)	C6—C7—C8—C9	0.0 (3)
C1—C2—C3—C4	0.5 (2)	C7—C8—C9—C10	−0.1 (3)
C2—C3—C4—C5	−176.49 (17)	C7—C8—C9—N2	−179.10 (16)
C2—C3—C4—S1	−0.2 (2)	O1—N2—C9—C10	9.7 (3)
C1—S1—C4—C3	−0.06 (15)	O2—N2—C9—C10	−170.11 (18)
C1—S1—C4—C5	176.43 (16)	O1—N2—C9—C8	−171.17 (17)
C6—N1—C5—C4	−178.81 (16)	O2—N2—C9—C8	9.0 (3)
C3—C4—C5—N1	−179.32 (19)	C8—C9—C10—C11	−0.6 (3)
S1—C4—C5—N1	4.7 (3)	N2—C9—C10—C11	178.44 (16)
C5—N1—C6—C11	−37.0 (3)	C9—C10—C11—C6	1.3 (3)
C5—N1—C6—C7	146.04 (18)	C7—C6—C11—C10	−1.4 (3)
C11—C6—C7—C8	0.8 (3)	N1—C6—C11—C10	−178.30 (17)

### Hydrogen-bond geometry (Å, º)

**Table:**

*D*—H···*A*	*D*—H	H···*A*	*D*···*A*	*D*—H···*A*
C1—H1···O2^i^	0.95	2.50	3.412 (2)	160
C2—H2···N1^ii^	0.95	2.62	3.556 (2)	169

Symmetry codes: (i) *x*−1, −*y*+1, *z*−1/2; (ii) −*x*+1/2, *y*−1/2, −*z*+1/2.
